# Development and comparison of an esophageal Doppler monitoring-based treatment algorithm with a heart rate and blood pressure-based treatment algorithm for goal-directed fluid therapy in anesthetized dogs: A pilot study

**DOI:** 10.3389/fvets.2022.1008240

**Published:** 2022-10-03

**Authors:** Inken Sabine Henze, Laura Hilpert, Annette P. N. Kutter

**Affiliations:** Section of Anesthesiology, Department of Clinical Diagnostics and Services, Vetsuisse Faculty, University of Zurich, Zurich, Switzerland

**Keywords:** canine, fluid responsiveness, goal-directed fluid therapy (GDFT), hypotension, esophageal Doppler monitor (EDM)

## Abstract

The objective of this pilot study was to determine the feasibility of a study comparing the efficacy of an esophageal Doppler monitor (EDM)-based fluid therapy algorithm with a heart rate (HR)- and mean arterial blood pressure (MAP)-based algorithm in reducing hypotension and fluid load in anesthetized dogs. Client-owned dogs undergoing general anesthesia for surgical procedures were randomized to two groups. An EDM probe for monitoring blood flow in the descending aorta was placed in each dog before receiving a crystalloid bolus (5 mL/kg) over 5 min. Fluids were repeated in case of fluid responsiveness defined by increasing Velocity Time Integral (VTI) ≥ 10% in group EDM and by decreasing HR ≥ 5 beats/min and/or increasing MAP ≥ 3 mmHg in group standard. The feasibility outcomes included the proportion of dogs completing the study and the clinical applicability of the algorithms. The clinical outcomes were the total administered fluid volume and the duration of hypotension defined as MAP < 60 mmHg. Data was compared between groups with Mann-Whitney *U*-test. *p* < 0.05 were deemed significant. Of 25 dogs screened, 14 completed the study (56%). There were no differences in the proportion of recorded time spent in hypotension in group standard [2 (0–39)% (median (range))] and EDM [0 (0–63) %, *p* = 1], or the total volume of fluids [standard 8 (5–14) mL/kg/h, EDM 11 (4–20) mL/kg/h, *p* = 0.3]. This study declined the feasibility of a study comparing the impact of two newly developed fluid therapy algorithms on hypotension and fluid load in their current form. Clinical outcome analyses were underpowered and no differences in treatment efficacy between the groups could be determined. The conclusions drawn from this pilot study provide important information for future study designs.

## Introduction

Arterial hypotension is a frequent complication of general anesthesia in both human (1) and veterinary patients (2, 3). In people, post-anesthetic mortality and organ dysfunction have both been associated with the incidence (4) and the duration (5, 6) of intraoperative hypotension. A quick detection of the underlying cause of hypotension is crucial for specific treatment (6, 7), yet this remains challenging as neither measurements of cardiac output nor of vascular resistance are readily available in clinical settings. The administration of fluids has been a common approach to treat intraoperative hypotension, yet evidence grew that not only too little but also too much fluid administration can be detrimental for the patient. In people, the administration of large volumes of fluid has been reported to increase morbidity and mortality both in standard surgical and in critical patients (8, 9). In critically ill dogs, a significant association between percentage of fluid overload, illness severity and mortality has been demonstrated (10). The aim of perioperative fluid administration is to optimize the preload of the heart (11), to improve stroke volume (SV) (11, 12) and finally tissue perfusion (13, 14). For preload optimization, the concept of fluid responsiveness has been introduced, defined as a >10–15% increase in SV in response to a fluid bolus (15–18). In veterinary medicine, the concept of evaluating fluid responsiveness in clinical patients is still new, and only recently, many studies have evaluated a variety of non-invasive parameters to assess and predict fluid responsiveness in both clinical (15–24) and experimental settings (25, 26).

For measuring changes (Δ) of SV in clinical patients, a readily available, non-invasive method is required. A veterinary esophageal Doppler monitor (EDM) system is available for dogs down to a body weight of 0.5 kg. An EDM measures the blood flow velocity in the descending aorta (13), calculates the Velocity Time Integral (VTI) and displays it as stroke distance in cm. In people, VTI can be used as a surrogate for SV (27) and SV correlates strongly with VTI in dogs (28). The strength of the EDM is that the curve can be visually controlled and offers information not only about VTI but also measures mean acceleration (MA), peak velocity (PV), heart rate (HR) and duration of blood flow. The various EDM variables allow the anesthetist to estimate how the cardiovascular system is reacting to therapeutic interventions and to target fluid or vasopressor therapy during general anesthesia (29), which is the purpose of a goal-directed fluid therapy (GDFT) approach (30).

Clinically, Δ HR and Δ mean arterial blood pressure (MAP) are still used to evaluate fluid responsiveness, although they are reported to be unreliable (31). While the use of algorithms to guide the perioperative fluid plan has been highly recommended in human medicine (32), no GDFT approach using an algorithm has been suggested for veterinary patients.

Therefore, a pilot study was performed to assess the concept of a newly developed EDM-based GDFT algorithm compared to an algorithm based on the standard parameters Δ HR and Δ MAP. The feasibility outcomes included the proportion of dogs that completed the trial and the clinical applicability of the two newly designed algorithms including non-compliance to the algorithms. In addition, we aimed to compare the proportion of time spent in hypotension, the fluid load and the anesthetist's feeling of understanding the patients' cardiovascular state between the groups as clinical outcomes. We hypothesized that both hypotensive periods and fluid load would be reduced when using an algorithm based on Δ VTI and that the anesthetist's feeling of understanding the patients' cardiovascular state would be increased in group EDM.

## Materials and methods

### Design

This single-site, prospective, randomized, clinical pilot study was conducted at the Veterinary Hospital of the University of Zurich. Approval was granted by the ethical committee of the canton Zurich (ZH037/20). Written informed owner consent was obtained for each animal before inclusion in the study.

### Animal enrollment

**Figure 1:**
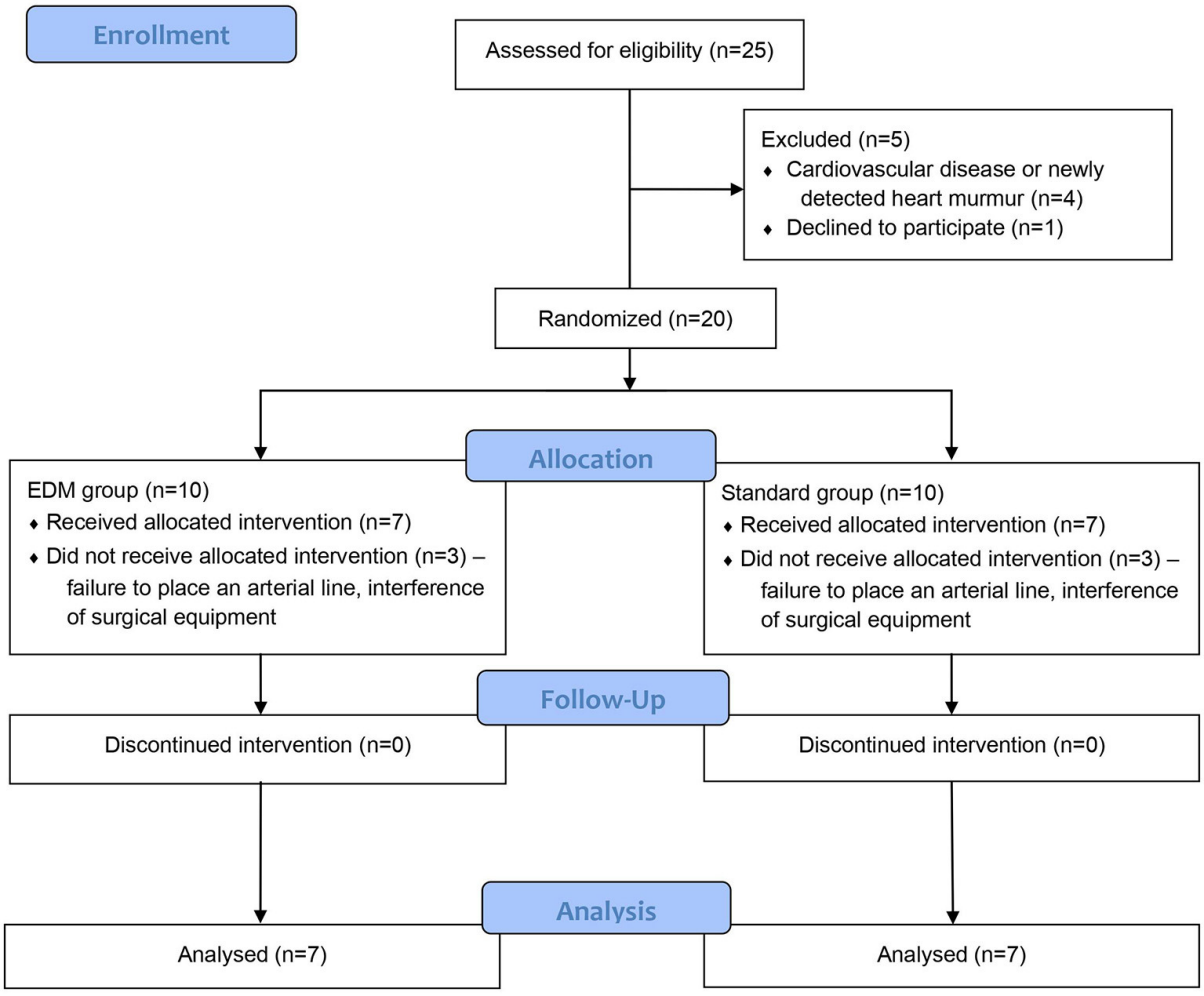
CONSORT flow diagram of veterinary patient enrollment and outcomes. Group esophageal Doppler monitor (EDM): fluid management during anesthesia was conducted according to an algorithm based on EDM variables. Group standard: fluid management during anesthesia was conducted according to an algorithm based on the standard parameters heart rate and mean arterial blood pressure.

Client-owned dogs of either sex undergoing anesthesia of at least 1 h duration could be included. The study was performed according to the Consolidated Standards of Reporting Trials (CONSORT) statement (Figure 1). The dogs were randomly assigned to either group standard or group EDM using the website www.randomization.com (last accessed 20.12.2021). If a dog needed to be excluded, the next dog was allocated to the excluded dog's group. Pre-established exclusion criteria comprised any esophageal pathology, interventions involving the oral cavity or esophagus, a heart murmur noticed during physical examination, or a known cardiac disease. The first reason for the latter was the study protocol including the administration of at least one fluid bolus. The second reason was that the hemodynamic effect of a fluid challenge could vary leading to a response that might result in a different effect on SV, compared to a healthy patient with the same preload.

### Anesthesia and instrumentalization

The anesthetic regimen for each dog was chosen individually, based on preanesthetic clinical examination, medical history, and planned intervention. All cases were managed by the same anesthetist. Food, but not water, was withheld for at least 6 h. If sedation was required for placement of an intravenous (IV) catheter of appropriate size (VasoVet, B. Braun Medical AG, Switzerland), the dogs received an intramuscular (IM) injection with 20–30 μg/kg acepromazine (Prequillan, Arovet AG, Switzerland), and/or 2–8 μg/kg medetomidine (Medetor, Virbac AG, Switzerland) or 5 μg/kg dexmedetomidine (Dexdomitor, Provet AG, Switzerland). If a catheter was already in place, but the dog nervous, anxious, or excited so that sedation was deemed necessary to ensure a smooth induction of general anesthesia, 0.3–4 μg/kg dexmedetomidine was injected IV. As part of premedication, every dog received 0.2 mg/kg methadone either IV or IM. General anesthesia was induced with either 1 mg/kg propofol (Propofol 1% MCT Fresenius, Fresenius Kabi, Switzerland) IV and titrated to effect with additional boli of 0.5 mg/kg or with 0.5 mg/kg alfaxalone (Alfaxan Multidose, Dr. E. Graeub, Switzerland) IV, followed by boli of 0.25 mg/kg IV. If possible, a co-induction with 1 mg/kg ketamine (Ketanarkon, Streuli Pharma, Switzerland) IV was performed. The type, dose and route of premedication and induction drugs for each dog are reported in the results section.

After securing the airway with a cuffed endotracheal tube of appropriate size (Super Safety Clear, Teleflex Medical, Switzerland), general anesthesia was maintained with isoflurane (IsoFlo, Zoetis Schweiz, Switzerland) or sevoflurane (Sevorane, AbbVie Deutschland GmbH & Co. KG, Germany) to effect in oxygen and air (initial FIO_2_ 50%) *via* a circle system mounted on an anesthesia machine (Aespire View, Anandic, Switzerland). A fentanyl infusion (1–13 μg/kg/h; Fentanyl, Sintetica SA, Switzerland) was given IV for intraoperative analgesia. Both the doses of the inhalational anesthetic and fentanyl were adjusted to effect based on the algorithms described below. A crystalloid infusion was administered IV at a rate of 5 mL/kg/h (Plasma-Lyte A, Baxter AG, Switzerland).

All dogs were initially left to ventilate spontaneously. If apnea or hypoventilation, defined by an end-tidal CO_2_ (PE'CO_2_) > 50 mmHg (>6.67 kPa), occurred, mechanical ventilation was initiated with a volume- or pressure-controlled ventilation mode to obtain a tidal volume of 10 mL/kg. The respiratory rate was set to obtain a PE'CO_2_ between 35 and 45 mmHg (4.67–6.0 kPa).

Immediately following induction of general anesthesia, cardiorespiratory monitoring was installed in each animal comprising anesthetic gas analysis, capnography, electrocardiogram, pulse oximetry, non-invasive blood pressure and esophageal temperature measurement (Cardiocap/5, GE Datex-Ohmeda, Switzerland). In all dogs, aseptical placement of an arterial 22-gauge catheter (Surflo, Terumo Europe, Belgium) into a metatarsal artery for invasive measurement of systemic arterial blood pressures *via* a transducer (DTXPlus; Argon Medical Devices, Netherlands) was attempted. The catheter was connected to the transducer through a non-compliant, fluid filled extension line. Transducers were zeroed to atmospheric pressure and leveled at the base of the heart.

In addition, an EDM was installed in each dog after positioning for surgery inside the operating theater to enable retrospective evaluation of fluid responsiveness and EDM values for all dogs (CardioQ ODM V+ Veterinary Monitor, Deltex Medical Ltd., UK). The EDM probe (EDP240 Doppler Probe, Deltex Medical, SC, USA) was connected to the EDM monitor and insertion depth was externally estimated by holding the probe next to the dog with the tip midway between the heart and the assumed location of the diaphragm. After a lubricant had been applied to the tip of the probe, it was inserted into the esophagus and advanced until resistance from the cardia was felt. An optimal signal was searched by slow rotation and retraction of the probe with auditory help from the device. At optimal positioning, a characteristic high-pitch pulsatile sound can be heard, and the displayed curve should be of sharp triangular shape with a spike at peak velocity. The probe was carefully attached to the endotracheal tube to ensure stable positioning. The number of cycles used to calculate the individual variables was adjusted to ten. As soon as a good Doppler signal had been obtained, data collection was started. All parameters were recorded every 5 min and blood pressures and HR were recorded every minute.

### Outcome measures

The feasibility outcomes included the recruitment rates and proportion of dogs that completed the trial and the clinical applicability of the two newly designed algorithms, including the non-compliance to these. The measures taken by following the algorithms were retrospectively evaluated for their appropriateness and clinical feasibility.

**Figure 2:**
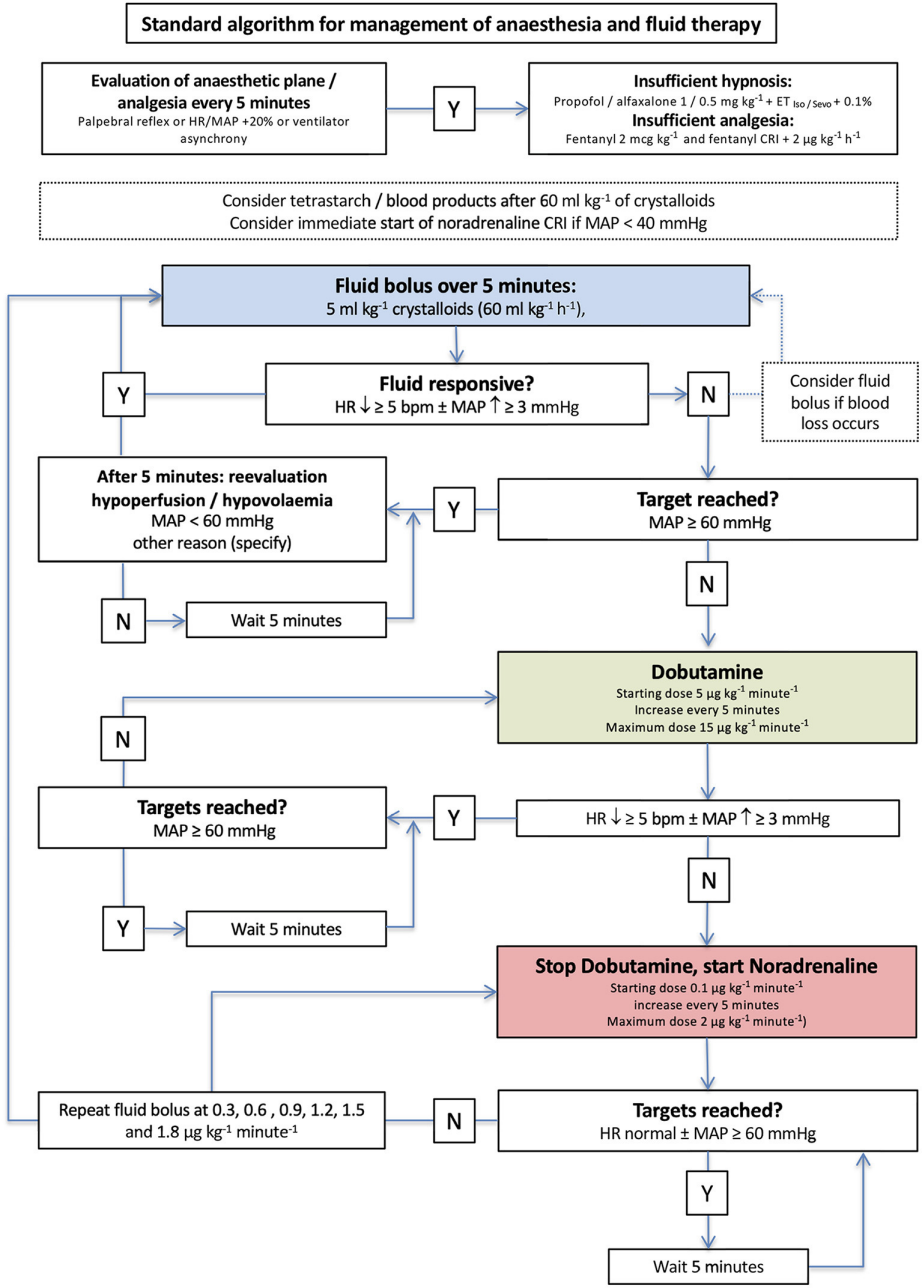
Algorithm for management of anesthesia and goal-directed fluid therapy in group standard. The initial crystalloid bolus of 5 ml/kg was administered to every dog. Y, yes; N, no; iso, isoflurane; sevo, sevoflurane; IV, intravenous; CRI, continuous rate infusion; bpm, beats per minute.

**Figure 3:**
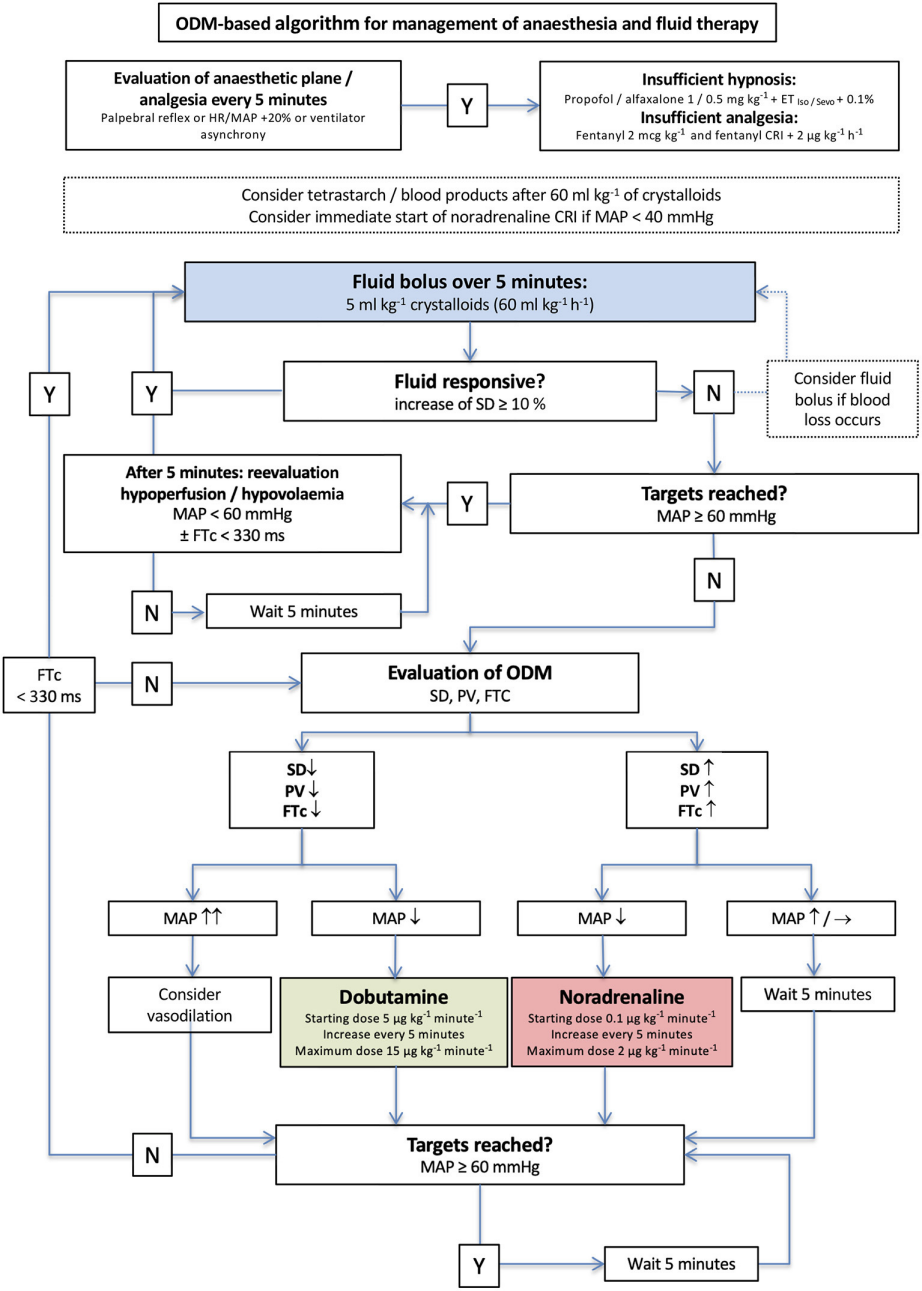
Algorithm for management of anesthesia and goal-directed fluid therapy in group esophageal Doppler monitor (EDM). The initial crystalloid bolus of 5 ml/kg was administered to every dog. Y, yes; N, no; iso, isoflurane; sevo, sevoflurane; IV, intravenous; CRI, continuous rate infusion; bpm, beats per minute.

The clinical outcome measures and evaluation of the methods are described as follows: hypotension was defined as MAP < 60 mmHg and managed based on two different algorithms in group standard (Figure 2) and group EDM (Figure 3) that had been designed by the authors before the pilot study was started.

Baseline parameters were recorded in both groups: HR, systolic arterial blood pressure (SAP), diastolic arterial blood pressure (DAP), MAP, quality of EDM signal, VTI, PV, MA, and corrected flow time (FTc). Immediately thereafter, one initial fluid bolus of 5 mL/kg (Plasma-Lyte A, Baxter AG, Switzerland) was administered over 5 min independent of the recorded parameters. Care was taken not to coincide the bolus with the beginning of surgical manipulation or administration of additional drugs.

Straight after completion of the fluid bolus and 1 min later the parameters were recorded. For the assessment of fluid responsiveness, values for HR and MAP, or VTI 1 min after completion of the bolus administration were used.

In group standard, a dog was considered fluid responsive if HR decreased ≥5 beats/min and/or MAP increased ≥ 3 mmHg (Figure 2). The anesthetist decided about therapy based on MAP every 5 min and was blinded to the recorded EDM variables (Figure 2). In dogs in group EDM, all EDM variables were visible to the anesthetist who decided about therapy based on MAP and EDM variables every 5 min (Figure 3). A dog was considered fluid responsive if the fluid bolus caused an increase in VTI ≥ 10% [(VTI_after_−*VTI*_before_)/VTI_before_] (Figure 3).

When an individual was considered fluid responsive, another fluid bolus of 5 mL/kg was administered and repeated until no more fluid responsiveness was noticed according to the predetermined criteria of each group (Figures 2, 3).

In case of a lacking fluid response with MAP < 60 mmHg in group standard, a dobutamine constant rate infusion (CRI) was started at a rate of 5 μg/kg/min (Figure 2). If a positive response was observed but values within the reference range not yet achieved, the dobutamine CRI was increased to 10 μg/kg/min and in a third step possibly to 15 μg/kg/min. If the dog did not respond to dobutamine, its administration was stopped and a noradrenaline CRI at 0.1 μg/kg/min was started.

When a dog in group EDM was non-responsive to either the initial or a repeated fluid bolus, MAP was assessed and if it was < 60 mmHg, the EDM variables VTI and FTc supported further decision making (Figure 3). If VTI was low and FTc <330 ms, a dobutamine CRI was started at a rate of 5 μg/kg/min. If an increase in VTI and FTc > 330 ms had been noticed accompanying a MAP < 60 mmHg, a noradrenaline CRI was started at 0.1 μg/kg/min and re-evaluated and increased by 0.1 μg/kg/min every five min if needed (Figure 3).

Any changes in the anesthetic management like administration of additional analgesics or a fentanyl bolus IV followed by an increase in the rate of the fentanyl CRI were noted as well as surgical stimuli. The time of induction of general anesthesia, start of surgery, completion of the surgical intervention, change in the dog's position, termination of general anesthesia and extubation were taken. The total amount of administered fluids was calculated. Data recording was completed earliest at 60 min after the initial fluid bolus and latest with termination of general anesthesia, and the EDM probe was removed before extubation.

For each dog, the proportion of recorded time spent in hypotension was calculated (recordings with MAP < 60 mmHg/all recorded MAPs).

After recovery of the dog, the anesthetist evaluated her overall feeling of understanding the dog's cardiovascular state during the anesthetic on a visual analog scale (VAS). On a line of 100 mm, one point was subjectively marked between 0 (no control at all) and 100 (absolute control at any time).

### Statistical methods

During the anesthetic, data was entered in an Excel sheet (Microsoft Office 2019, Microsoft Corporation, WA, USA) and statistical analysis of the clinical outcome parameters were compared between groups with Mann-Whitney U test using GraphPad Prism 9.2.0 (GraphPad Software Inc., CA, USA). A *p* < 0.05 was considered significant. The results are reported as the median and range, or as absolute numbers and percentages.

## Results

### Feasibility outcomes

Of the 25 screened dogs, 80% (20/25) were eligible for participation, and 56% (14/25) completed the study (Figure 1). Four dogs were excluded for not meeting the inclusion criteria, and for one dog, informed owner consent was not available. The remaining 20 dogs were randomized to either group standard or group EDM. Thereafter, six dogs were excluded either because no arterial catheter could be placed, or because the use of surgical equipment such as a trephine, arthroscopic shaver, or monopolar cautery highly interfered with the EDM signal. The other 14 dogs completed the current study. Both groups consisted of seven dogs each.

In one dog in group EDM, due to severe cardiovascular instability with MAP 38–48 mmHg, the algorithm was not followed exactly and dobutamine and noradrenaline CRIs were co-administered to effect (**Table 2**). As fluid boli were administered according to the algorithm, this dog was not excluded from the preliminary clinical outcome analysis.

### Baseline data

No difference was detected among groups for age, weight, American Society of Anesthesiologists (ASA) status, duration of anesthesia and duration of measurements (Table 1). In both groups, 2/7 dogs had an ASA status > 2. All dogs were mechanically ventilated during the measurements. The premedication of the dogs, their ASA status and the procedures are summarized in Table 2.

**Table 1:** Median and range of age, weight, duration of anesthesia, and duration of measurements in 14 clinical canine patients receiving intraoperative fluid therapy based on two different treatment algorithms [standard and esophageal Doppler monitor (EDM)].

**Variable**	**Group standard**		**Group EDM**		***p*-value**
	**Median**	**Range**	**Median**	**Range**	
Age (months)	39	8–122	107	7–120	0.38
Weight (kg)	12.2	4.5–35.3	13.2	7–22	0.8
Duration of anesthesia (minutes)	165	105–314	195	119–336	0.62
Duration of measurements (minutes)	70	59–82	71	59–266	0.74

**Table 2:** Individual description of 14 clinical canine patients receiving intraoperative fluid therapy based on two different treatment algorithms.

**Dog**	**Breed**	**Intervention**	**Route**	**ACP**	**Med**	**Dex**	**Pro/Alf**	**Ket**	**Nor/Dob**	**Fluid boli** **fluid response/all**	**% MAP < 60 mmHg**	**ASA**
**Group standard**				μ**g/kg**			**mg/kg**	**μg/kg/minute**			
2	German shepherd	Cryptorchid castration	IM	20	2		P 1.5	K 1		3/3	39	2
9	Skye terrier	Splenectomy, liver biopsy, castration	IM	20	5		P 0.5	K 1		0/1	0	2
11	Cross- Breed	Exploration of inguinal fistula	IM	20			P 1.5	K 1		0/2	9	2
17	Maltese	Diaphragmatic hernia, ovariectomy	IV				A 4.4	K 1	Dob 5	1/3	17	3E
18	Dachs- bracke	Laparoscopic ovariectomy	IM		5		P 1	K 1		2/3	32	1
19	French bulldog	Arthroscopy, tibial plateau leveling osteotomy	IM	30			P 5	K 1		0/1	0	3
20	Bolonka	Tibial plateau leveling osteotomy	IM	30			P 2.5	K 1		0/1	0	2
**Group EDM**			
1	Shiba Inu	Ahmed valve, enucleation	IM	20			P 1	K 1		2/4	0	2
5	Beagle	Ovariohysterectomy, mastectomy	IV			0.3	P 6	K 1		0/1	0	2
7	Border collie	Conjunctival flap	IM		8		P 2.5			0/1	0	2E
8	Longhaired collie	Perineal hernia	IV			1	P 3.2	K 1		2/4	0	2
12	Labrador retriever	Enterotomy due to a foreign body	IV				P 3	K 1	Nor 0.1–0.6	2/4	49	4E
14	Pug	Mast cell tumor excision	IV				P 3	K 1	Nor 0.2–0.6 & Dob 2–4	1/3	63	3
16	Cross- Breed	Tibial fracture, osteosynthesis	IV			4	A 1.5			5/6	25	2

The number of fluid boli evoking a fluid response are shown as a ratio of all administered fluid boli (“all”). Route, route of administration of premedication including methadone; IV, intravenous; IM, intramuscular; ACP, acepromazine; Med, medetomidine; Dex, dexmedetomidine; the following all administered IV: P(ro), propofol; A(lf), alfaxalone; K(et), ketamine; Dob, dobutamine; Nor, noradrenaline; MAP, mean arterial blood pressure; ASA, American society of anesthesiologists classification; E, Emergency.

### Clinical outcomes

Hypotension occurred in 4/7 (57%) dogs in group standard and in 3/7 (43%) dogs in group EDM. Hypotension ≥ 10 min occurred in 3/7 (43%) dogs in both groups. The percentage of time spent in hypotension was not significantly different between group standard with 2 (0–39)% [median (range)] and group EDM with 0 (0–63)% (*p* = 1, Table 2).

The total volume of administered fluids was not different between group standard [8 (5–14) mL/kg/h] and group EDM [11 (4–20) mL/kg/h] (*p* = 0.3). The dogs in group standard received 2 (1–3) boli while the dogs in group EDM received 4 (1–6) boli (*p* = 0.12). Of these boli 6/14 (43%) were judged as evoking fluid responsiveness in group standard, while in group EDM 12/23 (52%) boli evoked responses, all judged by the respective method (Table 2). In group standard, 3/7 dogs and in group EDM, 2/7 dogs only received one fluid bolus because they were not fluid responsive.

All variables before and after the boli with or without fluid responsiveness assessed with EDM are shown in Tables 3A–C. Of all 37 boli administered throughout the study, 20 evoked a fluid response in terms of Δ VTI ≥ 10% (54%).

**Table 3A:** Group standard.

**Variable**	**All group standard (*****n*** = **14)**					
	Δ **Velocity Time Integral 14% (**−**54–433%)**					
	**Before bolus**			**After bolus**		
	**Median**	**Min**	**Max**	**Median**	**Min**	**Max**
Heart rate (beats/minute)	67	40	133	73	46	153
Systolic AP (mmHg)	104	84	150	108	92	150
Diastolic AP (mmHg)	51	39	70	50	40	80
Pulse pressure (mmHg)	52	39	103	56	43	96
Mean AP (mmHg)	63	42	88	64	50	96
Velocity Time Integral (cm)	9.7	0.6	19.4	9.6	3.2	22.2
Minute distance (cm)	555	40	1,450	855	221	1,928
Peak velocity (m/s)	72	13	153	73	31	154
Mean acceleration (m/s)	8.3	2.4	25.4	9.4	2.5	22.2
Flow time corrected (ms)	187	103	315	236	107	368						
**Variable group standard**	**Group Standard (*****n*** = **8)** Δ **Velocity Time Integral** ≥**10%**, **27% (10–433%)**						**Group standard (*****n*** = **6)** Δ **Velocity Time Integral**<**10%**, **7% (**−**25–5%)**					
	**Before bolus**			**After bolus**			**Before bolus**			**After bolus**		
	**Med**	**Min**	**Max**	**Med**	**Min**	**Max**	**Med**	**Min**	**Max**	**Med**	**Min**	**Max**
Heart rate (beats/minute)	67	40	133	67	46	153	68	45	92	84	68	141
Systolic AP (mmHg)	101	84	140	105	98	150	110	98	150	114	92	137
Diastolic AP (mmHg)	51	39	70	47	43	63	50	43	68	52	40	80
Pulse pressure (mmHg)	48	39	88	56	52	96	53	46	103	54	43	84
Mean AP (mmHg)	61.5	42	84	63	56	77	66	56	88	67	50	96
Velocity Time Integral (cm)	7.25	0.6	18.9	9	3.2	22	7.1	4.2	19.4	9.6	3.6	18.7
Minute distance (cm)	431	40	1,450	667	221	1,928	438	313	1,300	916	245	1,515
Peak velocity (m/s)	72.5	13	153	86.5	31	154	72	38	122	73	38	120
Mean acceleration (m/s)	7.0	2.4	25.4	8.8	3.6	22.2	8.3	4.6	14.7	10.2	2.5	13.6
Flow time corrected (ms)	179	103	315	220	107	337	194	170	269	194	170	269

Median (med) and range of cardiovascular variables measured before and after 14 intravenous (IV) boluses of 5 mL/kg Plasma-Lyte A in 7 clinical canine patients receiving intraoperative fluid therapy based on a blood pressure and heart rate based (standard) algorithm. Data for all boli are displayed in the upper table, and data for boli divided into fluid responsive and not fluid responsive by Δ Velocity Time Integral assessed with EDM are displayed in the lower table.

**Table 3B:** Group EDM.

**Variable**	**All Group EDM (*****n*** = **23)**					
	Δ **Velocity Time Integral 10% (**−**31–46%)**					
	**Before bolus**			**After bolus**		
	**Median**	**Min**	**Max**	**Median**	**Min**	**Max**
Heart rate (beats/minute)	76	41	162	81	49	162
Systolic AP (mmHg)	109	71	138	111	71	138
Diastolic AP (mmHg)	57	50	66	60	50	67
Pulse pressure (mmHg)	50	15	72	50	15	73
Mean AP (mmHg)	69	61	82	72	61	82
Velocity Time Integral (cm)	10.1	7.2	18.7	11.2	7.7	16.4
Minute distance (cm)	782	410	2,281	869	454	2,415
Peak velocity (m/s)	76	40	109	78	42	99
Mean acceleration (m/s)	8.5	2.7	18.4	9.3	2.7	18.4
Flow time corrected (ms)	276	165	541	292	148	541						
**Variable**	**Group EDM (*****n*** = **12)** Δ **Velocity Time Integral** ≥**10%**, **19% (11–46%)**						**Group EDM (*****n*** = **11)** Δ **Velocity Time Integral**<**10%**, −**1% (**−**31–9%)**					
	**Before bolus**			**After bolus**			**Before bolus**			**After bolus**		
	**Med**	**Min**	**Max**	**Med**	**Min**	**Max**	**Med**	**Min**	**Max**	**Med**	**Min**	**Max**
Heart rate (beats/minute)	74	45	162	81	52	162	85	41	146	81	49	150
Systolic AP (mmHg)	84	71	131	83	71	118	118	79	138	129	91	138
Diastolic AP (mmHg)	57	50	65	57	50	65	59	55	66	63	54	67
Pulse pressure (mmHg)	27	21	69	27	15	63	54	15	72	67	37	73
Mean AP (mmHg)	67	61	78	70	61	82	72	65	82	78	67	82
Velocity Time Integral (cm)	9.1	7.2	11.9	11.6	8.5	15	12.2	8.5	18.7	10.4	7.7	16.4
Minute distance (cm)	726	410	1,928	839	582	2,248	839	582	2,248	1,066	454	2,415
Peak velocity (m/s)	68	40	85	73.5	42	99	85	70	109	81	65	98
Mean acceleration (m/s)	7.0	2.7	15.1	7.2	2.7	18	10.3	6.1	18.4	9.6	4.2	15.9
Flow time corrected (ms)	306	185	455	341	200	541	276	165	541	260	148	534

Median and range of cardiovascular variables measured before and after 14 intravenous (IV) boluses of 5 mL/kg Plasma-Lyte A in 7 clinical canine patients receiving intraoperative fluid therapy based on an esophageal Doppler monitor (EDM) algorithm. Data for all boli are displayed in the upper table, and data for boli divided into fluid responsive and not fluid responsive by Δ Velocity Time Integral assessed with EDM are displayed in the lower table.

**Table 3C:** Both groups.

**Variable**	Δ **Velocity Time Integral** ≥**10%, 22% (10–433%)** ***n*** = **20**						Δ **Velocity Time Integral**<**10%, 7% (**−**25–5%)** ***n*** = **17**					
	**Before bolus**			**After bolus**			**Before bolus**			**After bolus**		
	**Med**	**Min**	**Max**	**Med**	**Min**	**Max**	**Med**	**Min**	**Max**	**Med**	**Min**	**Max**
Heart rate (beats/minute)	71	40	162	73	46	162	79	41	146	82	49	150
Systolic AP (mmHg)	90	71	140	101	65	150	106	50	150	112	75	138
Diastolic AP (mmHg)	55	34	70	53	31	65	52	31	68	54	32	80
Pulse pressure (mmHg)	46	21	88	54	15	96	54	15	103	55	37	84
Mean AP (mmHg)	66	42	84	67	45	82	65	45	88	70	46	96
Velocity Time Integral (cm)	9.1	0.6	18.9	11.6	3.2	22	12.2	4.2	19.4	10.3	3.6	18.7
Minute distance (cm)	653	40	1,928	839	221	2,248	782	313	2,281	1,066	245	2,415
Peak velocity (m/s)	68	13	153	73.5	31	154	85	38	122	80	38	120
Mean acceleration (m/s)	7.0	2.4	25.4	7.9	2.7	22	9.7	4.6	18.4	9.8	2.5	15.9
Flow time corrected (ms)	247	103	455	275	107	541	254	165	541	254	138	534

Median and range of cardiovascular variables measured before and after 37 intravenous (IV) boluses of 5 mL/kg Plasma-Lyte A in 14 clinical canine patients under isoflurane or sevoflurane anesthesia receiving intraoperative fluid therapy based on two different treatment algorithms (standard and esophageal Doppler monitor (EDM)) divided into fluid responsive and not fluid responsive by Δ Velocity Time Integral assessed with EDM.

Δ Velocity Time Integral, (Velocity Time Integral after fluid bolus–Velocity Time Integral before fluid bolus)/Velocity Time Integral before fluid bolus in percent; min, minimum value; max, maximum value; AP, arterial blood pressure; pulse pressure, systolic–diastolic arterial blood pressure; minute distance = stroke distance × heart rate.

**Figure 4:**
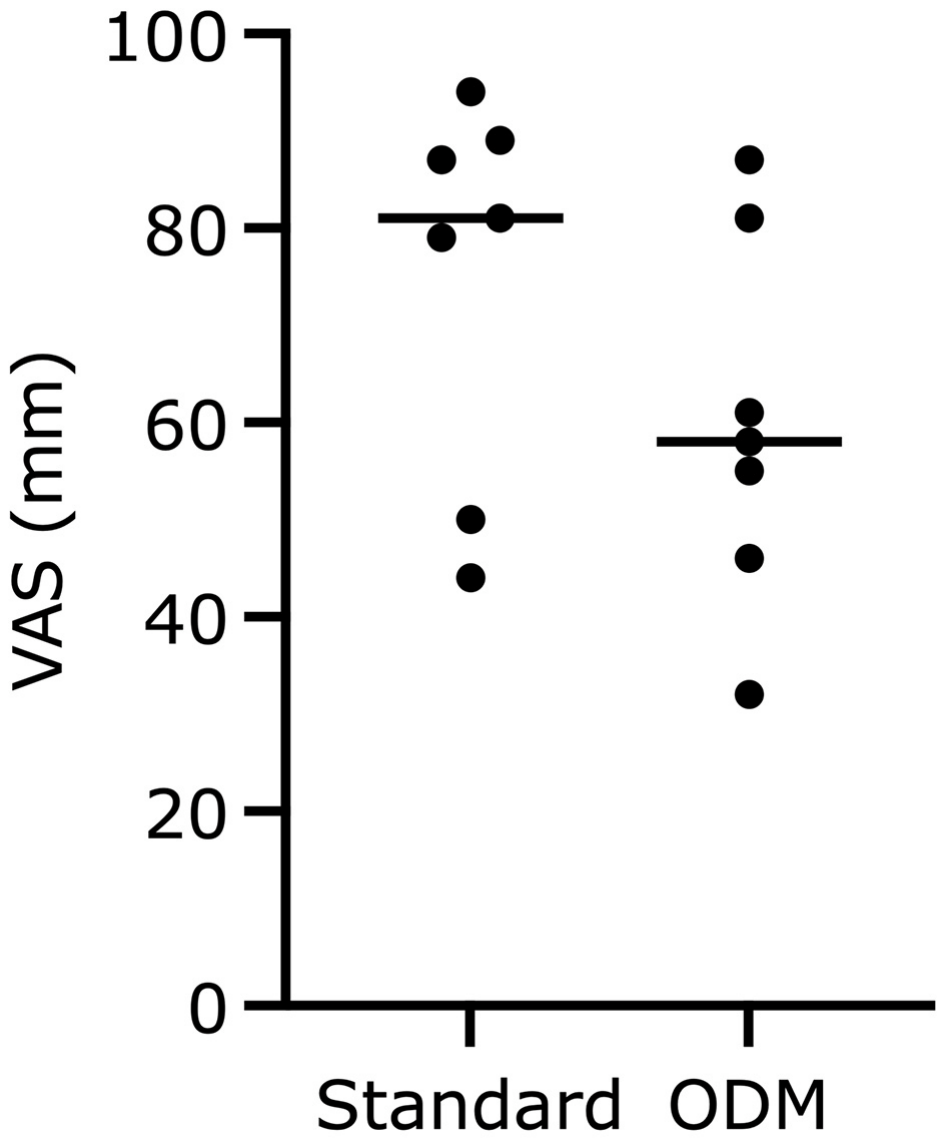
Scores on the visual analog scale (VAS) regarding the anesthetist's confidence to understand the dogs' cardiovascular state at any moment during general anesthesia. The median is marked by a horizontal line, respectively.

The VAS assessing the overall feeling of understanding the dogs' cardiovascular state was not different between group standard with 81 (44–94) mm and group EDM with 58 (32–87) mm (*p* = 0.25; Figure 4).

## Discussion

### Interpretation

To the authors' knowledge this is the first attempt to evaluate the feasibility of a study comparing two different GDFT algorithms in veterinary patients. As they had originally been designed for this pilot study and are the first of their kind, no data could be compared with those of other centers reporting similar trials.

Of the 25 screened dogs, 14 completed the study (56%). We consider this as low compared to the high workload for patient recruitment, preparation, expense for study material, and the need for a second person for data recording.

In a future large clinical trial in canine patients the low completion rate of only 56% in the current study and the overall incidence of hypotension ≥10 min (43%) need to be considered. To show a significant benefit of EDM over an algorithm based on standard parameters seems to be very challenging in a similar study design. For future studies, instead of excluding animals with cardiovascular disease as done in the current pilot trial, sole inclusion of critically ill animals with abnormal cardiovascular status should be considered. The authors suspect that in severely debilitated animals any additional knowledge of cardiac performance and vessel state could be more beneficial than in the currently examined patients. Furthermore, dogs undergoing procedures involving a trephine, arthroscopic shaver or monopolar cautery need to be excluded from the beginning because this equipment has been shown to cause artifacts during EDM measurements.

Following the algorithms designed for this study, all included dogs received one initial fluid bolus irrelevant of their MAP, similar to a previous veterinary study (21). Additionally, a bolus needed to be repeated when a fluid response was elicited also in normotensive animals. Many of the boli were administered even if MAP was within normal range, which possibly led to a more liberal fluid therapy than desirable. Yet it is not advisable to give fluids to a patient based on fluid responsiveness alone since a positive reaction to fluids is not equal to a need for fluids (14). In future studies evaluating GDFT algorithms, fluid treatment should be triggered by abnormal findings, e.g., hypotension, tachycardia and/or parameters indicating low SV.

Regarding the clinical outcomes, neither did the dogs in group EDM experience significantly shorter hypotensive periods, nor was the volume of administered fluids lower than in group standard. The availability of EDM parameters for anesthetic monitoring did not improve the anesthetist's overall feeling of understanding the dogs' cardiovascular state. The similar clinical outcomes between the groups need to be interpreted with caution though, as this pilot study was underpowered and the clinical results therefore inconclusive.

Nevertheless, we aim to discuss the scientific background available for the design of the study. In former studies in dogs, fluid responsiveness has been assessed after administration of a 3–20 mL/kg crystalloid fluid bolus over 1–15 min as either a > 10% decrease of HR and/or increase of MAP (33) or more recently as a ≥10–15% increase in SV or VTI (15–18, 20, 21, 24). The definition used in the current study in group standard (decrease of HR ≥ 5 beats/min and/or increase of MAP ≥ 3 mmHg) was chosen as we did not expect that a 5 mL/kg bolus of crystalloids would reduce a HR of 150 beats/min > 15 beats/min and/or increase MAP > 10%. We aimed to avoid the underestimation of fluid responsiveness by standard parameters, therefore we chose smaller differences in HR and MAP as indicators of a response contrary to those reported before. In group EDM, the definition of fluid responsiveness (increase in VTI ≥ 10%) was derived from the available literature using VTI as a surrogate for SV.

All over, 54% of the dogs had a Δ VTI ≥ 10% after a small bolus of 5 mL/kg. This was lower than the incidence of 76–100% found in earlier studies after administration of a bolus of 10 mL/kg in dogs anesthetized for abdominal surgery (15), of 15 mL/kg in dogs anesthetized for orthopedic procedures (20), or of 20 mL/kg in dogs anesthetized for ovariohysterectomy (21). One possible explanation for the lower fluid responsiveness is the smaller crystalloid bolus of 5 mL/kg, which was chosen to reduce the volume of fluids given to these clinical patients.

Another possible explanation for the lower fluid responsiveness in the current study might be the fact that 2/7 patients in each group were rated ASA 3 or 4 and were not considered healthy patients. In critically ill human patients with cardiovascular compromise, only 50% were reported to be fluid responsive (34). Similarly, fluid responsiveness was observed in 54% of anesthetized dogs with experimentally induced septicemia (26).

Hypotension occurred in 4/7 (57%) dogs in group standard and in 3/7 (43%) dogs in group EDM. Former studies in dogs displayed a lower incidence of hypotension of 38% (3) and 7% (2), respectively. In both studies, hypotension was defined as either MAP < 60 mmHg or SAP <80 mmHg irrespective of invasive or non-invasive blood pressure measurement. A large study in people demonstrated that the definition of hypotension has a major impact on its incidence (1). In contrast to the former studies, only invasive blood pressure measurement was used in our study and values were recorded and analyzed every minute. In people, invasive blood pressure measurement has been shown to detect twice as many hypotensive events during anesthesia as oscillometric measurement (35). In addition, Redondo et al. (3) analyzed the data of 1,281 anesthetics monitored by different anesthetists retrospectively based on standard anesthetic record sheets, while the current study and Gaynor et al. (2) assessed data prospectively. It can be suggested that the invasive, prospective, and frequent assessment of MAP in the current study led to a higher sensitivity and thus to a higher rate of detected values below the defined limit of 60 mmHg.

While in the formerly reported two studies only the incidence of hypotension was described, the duration of hypotension was calculated in the current study. The duration of hypotension relative to the total anesthesia time has only been described in one study comparing acepromazine to dexmedetomidine as preanesthetic agents in 341 dogs undergoing ovariohysterectomy ]37]. In that study, arterial blood pressure was measured non-invasively by oscillometric means and recorded every 5 min. The duration of hypotension was significantly higher after acepromazine with 25 (5–100) % than after dexmedetomidine with 17 (4–85) % (*p* = 0.04). The incidence of MAP < 60 mmHg was also higher (74%) in dogs after acepromazine than after dexmedetomidine (55%, *p* < 0.001), which is comparable to the incidence found in our study. Interestingly, in the current study in group EDM, only 1/7 dogs were premedicated with acepromazine vs. 5/7 dogs in group standard. Nevertheless, no difference in hypotensive time was found between groups [standard 2 (0–39)%, EDM 0 (0–63)%]. A trend to a wider range of hypotensive time and 60% more boli in group EDM can be seen (Tables 1–3A–C), although fewer dogs in group EDM received acepromazine. In the current study, acepromazine with its known vasodilative properties was only administered if deemed appropriate by the anesthetist, which is a possible confounder. It cannot be assessed retrospectively if the avoidance of acepromazine in 6/7 patients in group EDM was due to an expected increased risk of hypotension. On the other hand, if the patients in group EDM had an increased risk of hypotension and no difference in hypotension was found between the two algorithms, this could be explained either by the avoidance of acepromazine, by a benefit in group EDM that could not be shown due to differences among groups, or by the statistical inconclusiveness in this pilot study.

Similar to the current study, in all three previous studies presenting incidence of hypotension (2, 3, 36), a high variability in drugs used for premedication, induction, and maintenance of general anesthesia can be found. In future studies assessing GDFT algorithms at an early stage, a more standardized anesthetic protocol should be used to reduce possible confounding factors as different drugs might affect the results due to their various effects on the cardiovascular system. The original idea to test the algorithm in a variety of clinical patients should be delayed to a later phase in the evaluation process of GDFT algorithms.

The availability of EDM parameters for anesthetic monitoring did not improve the anesthetist's overall feeling of understanding the dogs' cardiovascular state. In group standard, the anesthetist had standard monitoring installed, which she had been used to for years. In group EDM, a new tool needed to be implemented and being paid attention to, which can be distracting and draw attention away from the known monitoring. Recognition of this somehow distracted attention might have led to a degree of insecurity. However, it might also be possible that dogs in group EDM were sicker overall, cardiovascularly more instable and thus had more challenging anesthetics. Retrospectively, it cannot be proven and is beyond the scope of this pilot study, if the additional monitoring tool or more challenging anesthetics led to the trend of lower VAS scores for the dogs in group EDM.

### Generalizability and limitations

The findings of this single-site pilot trial may not be generalizable to other veterinary institutions with a possibly different patient spectrum. Additionally, there was a subjective component to assessing the clinical feasibility of the two algorithms. The experienced anesthetist stated that she would have decided differently to what was required by following the algorithms in a few cases. Subjectively, the use of cardiovascularly active drugs might have been delayed compared to a more individual approach, and more fluids were administered than if judged by normal clinical assessment. While in group EDM, the algorithm allowed to select either dobutamine or noradrenaline at the same stage, this was not possible following the algorithm for group standard in its current version, which is not ideal for cases of hypotension resulting from relative hypovolemia and should be corrected in future algorithms. Considering that during general anesthesia, hypotension is often caused by drug-induced vasodilation and decreases in cardiac output, early adequate cardiovascular support with drugs targeting these issues under direct guidance by EDM parameters could improve veterinary patient care during clinical anesthesia (37).

In this first clinical study evaluating GDFT algorithms in anesthetized veterinary patients, several limitations were present. The use of 1 mg/kg ketamine for co-induction is common practice in our clinic to reduce the doses of short-acting induction drugs and their inherent side effects but might have been another confounding factor in the current study with effects on HR and MAP. The positive chronotropic and potentially positive inotropic and arrhythmogenic effects of ketamine might contribute to a lower incidence of hypotension with variable duration. For future study planning, we recommend not to use ketamine at all. Additionally, the measurements for the study were stopped after 60 min from the initial fluid bolus if anesthesia was stable, which might have led to a falsely high percentage of hypotensive time. Therefore, in a refined study design, all measurements should be conducted over the same time.

Another possible limitation was the use of Plasma-Lyte A, which contains acetate as a buffer. Large doses and fast administration rates are reported to induce vasodilation by metabolization of acetate to acetyl-coenzyme A and to lead to an increased nitric oxide (NO) synthesis (38). A bolus that itself induced vasodilation could have influenced the results and made correct interpretation of the dogs' reaction to a fluid bolus even more challenging. However, as both NO release and an optimized preload would decrease systemic vascular resistance, it was not possible to retrospectively assess if any bolus of Plasma-Lyte decreased the vessel tone more than usual, given the confounding effects the used anesthetic agents might have had on the dogs' cardiovascular system.

Due to the clinical nature of our study, VTI was measured with EDM, and the validity of the measured values could not be confirmed by a more invasive and more accurate method to measure SV. We do not know if the measured changes in EDM parameters were reliably detecting SV changes caused by therapeutic interventions although VTI has been shown to correlate strongly with SV in dogs (28). However, many of the previously published fluid responsiveness studies in dogs used transesophageal echocardiographic monitoring with or without assessment of the aortic area as the reference method (15, 16, 19, 20).

Fluid responsiveness in group standard was defined by different means compared to fluid responsiveness in group EDM. Comparing a parameter that has been defined in a different way in the two study groups might carry some bias in itself. In addition, the algorithm for group standard defined fluid responsiveness by a difference in absolute numbers while the algorithm for group EDM defined fluid responsiveness by a difference in percentage. Therefore, an EDM probe had been placed in all dogs, and an additional person documented the EDM parameters in group standard, which were inaccessible for the anesthetist. Retrospectively, the number of fluid boli evoking a response was compared when either measured with the definition for the respective group, or when assessed with Δ VTI (Tables 3A–C). Since the clinical outcome parameter of this study was incidence of hypotension, and the statistical results of this underpowered pilot trial should be considered as inconclusive, all comparisons of fluid responsiveness are only presented in descriptive statistics and no further comparative analysis of fluid responsiveness between groups was performed.

In this pilot study, influencing factors like size of the dog, underlying medical problem, anesthetic protocol, positioning, and type of surgery on the reliability of the EDM might not have been detected. Movement of the animal and stimuli due to surgical manipulation might have influenced all parameters. Chest movements due to mechanical ventilation as well as heart movements could all have affected ideal alignment of the probe's tip with the aortic blood flow over time before a less-than-ideal alignment would have been detected. Surgeries at the neck, intervertebral disc surgeries in which a trephine was used, arthroscopic shaving of intraarticular cartilage as well as the use of monopolar cautery highly interfered with the EDM signal and could not be used. However, as the EDM is considered a clinical tool, an evaluation during general anesthesia for surgeries seems ideal to assess and describe its utility in daily practice.

The two different treatment algorithms used in this study were designed by the authors explicitly for this study to assess their suitability for a larger clinical trial. Like in modern human studies we did not compare our algorithm to a group in which fluids were given liberally or without following any GDFT, but we compared two similarly built algorithms just differing in the monitoring method. In the preliminary results, no benefit of one algorithm over the other was found assessing duration of hypotension and fluid administration. This is in accordance with some studies in people comparing EDM guided fluid therapy to restrictive fluid therapy that found no extra value of EDM (39, 40), yet, due to the underpowered nature of the pilot study, no premature conclusions should be drawn.

This pilot study revealed major necessary modifications to the protocol before conducting a larger clinical trial. A future main study with a calculated sample size is required for creating evidence, yet it cannot be advised to be conducted with the protocol presented in this pilot trial. An improved algorithm that would allow to choose between administration of fluids or cardiovascular drugs based on additional EDM variables influenced by contractility and vasomotor tone (i.e., PV, MA, and FTc) could reduce fluid load, improve the overall outcome, and accentuate the value of an EDM by predicting fluid responsiveness. In future algorithms, the use of both fluids and cardiovascular drugs should be triggered by measurements of HR, MAP, VTI, PV, MA, and/or FTc that are not within normal limits to further restrict the volume of administered fluids in a GDFT approach.

## Conclusions

This study declined the feasibility of a study comparing the impact of two newly developed fluid therapy algorithms on hypotension and fluid load in their current form. Clinical outcome analyses were underpowered and no differences in treatment efficacy between the groups could be determined. The conclusions drawn from this pilot study provide important information for future study designs.

## Data availability statement

The original contributions presented in the study are included in the article/supplementary material, further inquiries can be directed to the corresponding author.

## Ethics statement

The animal study was reviewed and approved by ZH Veterinaeramt Zuerich, Zollstrasse 20, CH-8090 Zurich, Switzerland. Written informed consent was obtained from the owners for the participation of their animals in this study.

## Author contributions

IH: study design, design of treatment algorithms, data collection and management, data analysis, and preparation of manuscript. LH: design of treatment algorithms, data collection and management, data analysis, and preparation of manuscript. AK: study design, design of treatment algorithms, data collection and management, data analysis, and critical revision of manuscript. All authors contributed to the article and approved the submitted version.

## Conflict of interest

The authors declare that the research was conducted in the absence of any commercial or financial relationships that could be construed as a potential conflict of interest.

## Publisher's note

All claims expressed in this article are solely those of the authors and do not necessarily represent those of their affiliated organizations, or those of the publisher, the editors and the reviewers. Any product that may be evaluated in this article, or claim that may be made by its manufacturer, is not guaranteed or endorsed by the publisher.
